# Resveratrol in Patients with Minimal Hepatic Encephalopathy

**DOI:** 10.3390/nu10030329

**Published:** 2018-03-09

**Authors:** Giulia Malaguarnera, Manuela Pennisi, Gaetano Bertino, Massimo Motta, Antonio Maria Borzì, Enzo Vicari, Rita Bella, Filippo Drago, Michele Malaguarnera

**Affiliations:** 1 Research Center “The Great Senescence”, University of Catania, 95126 Catania, Italy; antoniomaria.borzi@gmail.com (A.M.B.); michele.malaguarnera@gmail.com (M.M.); 2 Department of Biomedical and Biotechnological Science, University of Catania, 95123 Catania, Italy; f.drago@unict.it; 3 Spinal Unit of Cannizzaro Hospital, University of Catania, 95100 Catania, Italy; manuelapennisi78@gmail.com (M.P.); mottam@unict.it (M.M.); 4 Department of Experimental and Clinical Medicine, University of Catania, 95123 Catania, Italy; gaetanobertinounict@gmail.com (G.B.); enzodante@email.it (E.V.); 5 Department “G.F. Ingrassia”, Section of Neurosciences, University of Catania, 95123 Catania, Italy; rbella@unict.it

**Keywords:** depression, anxiety, resveratrol, ammonia, quality of life, minimal hepatic encephalopathy

## Abstract

Background: Minimal Hepatic Encephalopathy (MHE) is characterized by an impairment of social interaction, emotional behavior, sleep disorders, physical and mental symptoms, and diminished Quality of Life (QoL). The aim of our study is evaluating the potential liver health promoting a perspective of Resveratrol (RV) activities and evaluate whether RV treatment may improve health related quality of life (HRQL) and reduce depression and anxiety in patients with MHE. Methods: *W*e evaluated depression using the Beck Depression Inventory test, anxiety with State-trait anxiety inventory test, quality of life through SF-36 test, and ammonia serum levels in 70 MHE patients that were randomized into two groups. Results: In the comparison between RV group and placebo group we observed a decrease in Back Depression Inventory (BDI) (*p* < 0.001), in State-trait anxiety inventory (STAI) (*p* < 0.001), and improve in physical function (*p* < 0.001), in role physical (*p* < 0.05), in body pain (*p* < 0.05), in general health (*p* < 0.001), in vitality (*p* < 0.05), and in social function (*p* < 0.001). Conclusions: Resveratrol showed efficacy in the treatment of depression, anxiety, and ammonia serum levels, and improved the quality of life Of MHE patients.

## 1. Introduction

Minimal Hepatic Encephalopathy (MHE) is a frequent and common complication of cirrhosis, affecting nearly 80% of cirrhotic patients. Physical examination in MHE is often normal; patients may present subtle abnormalieties, interfering with executive function, including their working memory and orientation, impairment of social interaction, emotional behavior, sleep disorders, physical and mental symptoms, and diminished Quality of Life (QoL) [1].

This underrecognized and underdiagnosized syndrome often presents minimal changes in personality and in memory, intellectual function, concentration, and coordination.

These disorders impinge on the health related quality of life and have a negative prognostic value in relation to the occurrence of both bouts of overt hepatic encephalopathy and death [2].

Resveratrol (RV) is a natural phytoalexin, which is isolated from the roots of with hellebore (Veratrum grandiflorum Hoes). RV is present in two isoforms, cis/trans, and trans isomer is the biologically active one. It has been report that RV plays role as antinflammatory, antioxidants, antiviral, and exerts anticancer activities through many different mechanisms [3,4,5,6,7,8,9,10,11].

RV showed protective effect against neuro-inflammation inhibiting reactive oxygen species (ROS) production and mitogen-activated protein kinases (MAPK) signal transduction pathways, and also inactivating signalling pathways of both nuclear factor-kappa b (NF-κB) and silent mating type information regulation 2 homolog 1 (SIRT1) [12,13]. SIRT belongs to a protein family consisting of seven individual members, which are involved in cellular metabolism, mitochondrial function, and stress responses modulation [14].

The RV attracted the interest of researchers for the combined antidepressant and anti-inflammatory effects [11].

The aim of the current study was the evaluation of RV in liver health, in health related quality of life (HRQL), and in reduction of depression and anxiety in patients with MHE.

## 2. Patients and Methods

### 2.1. Study Design

Seventy-five patients with cirrhosis and minimal cognitive impairment regarding selective attention and executive function were recruited between May 2013 and November 2016 at Cannizzaro Hospital in Catania (Italy).

The diagnosis of cirrhosis was based on case history, clinical examination, biochemical, endoscopic, and ultrasounds finding; or, when needed, on liver biopsy.

This observational study was approved by the research ethics committee of Cannizzaro Hospital.

All of the eligible patients were approached and asked to participate in the study; those who accepted signed the written the informed consent after reading carefully the patients information leaflet.

**Figure 1:**
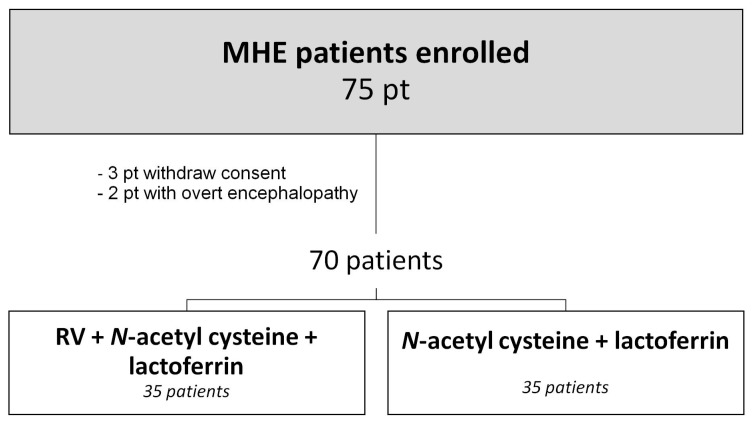
Study design. MHE = Minimal Hepatic Encephalopathy; RV=Resveratrol.

75 patients with diagnosis of MHE were enrolled in the study: three withdrew consent, two overt hepatic encephalopathy (Figure 1). The 70 patients were randomized into two groups, using permuted-block randomization with an allocation ratio 1:1 and block size of four. Random numbers were assigned according to the sequency of their inclusion and patients received respective study products. Group A were treated with RV in pills 19.8 mg, *N*-acetyl cysteine 600 mg, lactoferrin 23.6 g (Resvis Alfa-sigma, Bologna, Italy). Group B were treated with placebo: *N*-acetyl cysteine 600 mg, lactoferrin 23.6 g. The diagnosis of HE was made on the evaluation of consciousness, intellectual behavior, and neuromuscular functions. Mental status was assessed and graded on the basis of West-Haven Criteria [15]. The schedule was to receive for 90 days a supply of either RV or placebo. The measurement was made every month, both for efficacy and for tolerability tests.

### 2.2. Protocol

The inclusion criteria were age 25–65 years.

Cirrhotic patients who performed abnormally on psychometric tests but performed normally on clinical neuropsychiatric examination were diagnosed with MHE. MHE was diagnosed according to guidelines from the final report of the Working Party at the 11th World Congress of Gastroenterology in Vienna in 1998.

The exclusion criteria were: pregnancy and breast feeding; past history of overt hepatic encephalopathy (OHE); use of interferon or psychoactive drugs, such as benzodiazepines, psychotropic drugs, antiepileptic; alcohol abuse, history of diabetes or renal impairment; significant head injury; arterial hypertension. Patients’ characteristic included educational status, Child-Turcotte Pugh class, and etiology of cirrhosis [16].

Male patients were considered to have alcohol related cirrhosis if their alcohol intake was more than 50 g and female patients if their intake was more than 30 g for more than five years and if testing showed no viral metabolic or immunologic cause. Chronic hepatitis B and C were diagnosed when testing was positive for the viral markers Hepatitis B surface Antigen (HBsAg) and Hepatitis C virus antibody (anti-HCV) respectively.

All of the patients underwent to Psychometric Hepatic Encephalopathy Score (PHES) [17,18], Short Form 36 Health Survey (SF-36) [19,20,21,22], Beck Depression Inventory (BDI) [23], and State-Trait Anxiety Inventory (STAI) [22].

#### 2.2.1. Diagnosis of MHE

The use of neuropsychometric battery tests have been limited to research of minimal HE. We performed the Psycometric Hepatic Encephalopathy Score (PHES), which consists of five psychometric tests that measure psychomotor speed and precision, visual perception, visus-spatial orientation, visual construction, concentration, attention, and memory [17,18].

#### 2.2.2. Neuropsychological Testing

##### PHES Test Battery

PHES Scores a neuropsychological battery composed of five different tests: Number connection test A (NET-A) and B (NET-B), line tracing test, serial dotting test, and digit symbol test [15,16]. The test detects attention and psychomotor speed, areas that were most affected by HE. PHES score is obtained based on sum of the result of each test [17,18].

#### 2.2.3. SF-36 (Short Form—36 Health Survey)

SF-36 questionnaire measures with eight domain scales and 36 items, health status. [17]

SF-36 provides two summary scores: physical component and mental component and eight health status scales: Physical functioning, role limitation due to physical problems, bodily pain, General health perceptions, vitality, social functioning, role limitation due to emotional problems, and mental health.

Patients completed the questionnaire and the resulting scores were transformed onto a scale from 0 (the worst possible score) to 100 (the best possible score), as recommended by questionnaires originators [19,20,21,22].

#### 2.2.4. Beck Depression Inventory (BDI)

BDI is the most widely used instrument for detecting depression. It is a 21-item self report rating inventory that measures characteristic attitudes and symptoms of depression, including mood, sense of failure, pessimism, guilt, punishment, self-dissatisfaction, self dislike, self accusation, crying, suicidal ideas, irritability, social withdrawal, indecisiveness, body image change, and work difficulty. Each answer is scored from 0 to 3. Higher total score indicate more severe depressive symptoms. Index score of ≤9 is considered to be within normal range, a score of 10–15 shows minimal depressive symptomatology, a score of 16–31 points toward mild depression, a score of 32–47 is in favor of moderate depression, and a score of >47 indicates severe depression [23]. The maximum total score is 63, whereas the minimum is 0.

#### 2.2.5. State-Trait Anxiety Inventory (STAI)

Patients’ anxiety was measured using the 20-items state-trait anxiety inventory (STAI). Each of 20 items related to anxiety was scored as 1 (not at all), 2 (somewhat), 3 (moderately), 4 (very much) with higher score indicating more anxiety.

The scale is scored by summing the 20 responses (range 20–80) [24].

#### 2.2.6. Clinical and Laboratory Assessment

Medical history and physical examination had been performed for all patients. Laboratory tests included renal and liver function test, albumin, α fetoprotein, hemogram, prothrombin time index, fasting blood sugar, and venous ammonia concentration. The ammonia was determined according to the enxymatic determination of ammonia with glutamate dehydrogenase in a rapid and interference-free photometric determination (340 nanometer) of NH_4_^+^ in native blood plasma as according to De Fonseca-Wollheim method [25]. Blood was immediately taken by refrigerated transport sent to the laboratory for determination within 15 min from blood sampling. To stage cirrhosis Child-Pugh score was carried out.

Cirrhosis and Viral etiology were evaluated, considering alcohol consumption for cirrhosis and measuring through ELISA HBsAg levels and anti HCV levels for viral tests. When indicated, medical examination for autoimmune liver disease, Wilson disease, hemochromatosis, and Budd-Chiari Syndrome were performed.

#### 2.2.7. Neurological Assessment

Detailed neurological, psychiatric and mental state assessment had been performed to avoid undiagnosed psychiatric disorders.

#### 2.2.8. Efficacy and Tolerability Assessment

The primary efficacy measures were changed at the beginning and at the end of the study period in aspartate aminotransferase (AST), alanine aminotransferase (ALT), gamma-glutamyl-transpeptidase (γ-GT), albumin, alkaline phosphatase (ALP), prothrombin time, and ammonia.

Laboratory assessments included hemochrome, glycemia, creatininemia, and blood urea were monitored at baseline and monthly until the end of the trial. Each subject of both the groups underwent ultrasonography (US) examination of the liver, electrocardiography, and blood pressure with the use of standard techniques [26].

### 2.3. Statistical Analysis

Descriptive statistics, chi-square tests (for categorical variables), and *t*-tests (for continuous variables) were used to compare demographic and clinical characteristics. *p* values ≤ 0.05 were considered to be statistically significant. Means, actual numbers, and percentages were used to describe data. Statistical Analyses were performed by two-way analysis of variance (ANOVA), as well as by controlling for Bonferroni’s multiple correction.

## 3. Results

Demographics characteristics were similar between the two groups at baseline. At enrolment, no significant differences were observed in biohumoral tests, at etiologic factors in cirrhosis (Table 1).

### 3.1. Effects of RV on Biohumoral Findings

In the group that were treated with RV, we observed a significant decrease (*p* < 0.001) in urea, in NH4, in AST, in ALT, and γ-GT.

In the placebo group we observed a decrease (*p* < 0.05) in AST and ALT.

The comparison between RV group and placebo group shows a significant decrease in NH4 (*p* < 0.001) in AST (*p* < 0.001) and in ALT (*p* < 0.001) (Table 2).

### 3.2. Effects of RV in Depression, in Anxiety and in Quality of Life

Anxiety and quality of life. The comparison between before and after RV treatment showed a decreased in BDI *p* < 0.001 in STAI (*p* < 0.001) and increased in physical function (*p* < 0.001), in role physical (*p* < 0.05), in Body pain (*p* < 0.001), in general health (*p* < 0.001), in vitality (*p* = 0.002), and in social function (*p* < 0.001).

**Figure 2:**
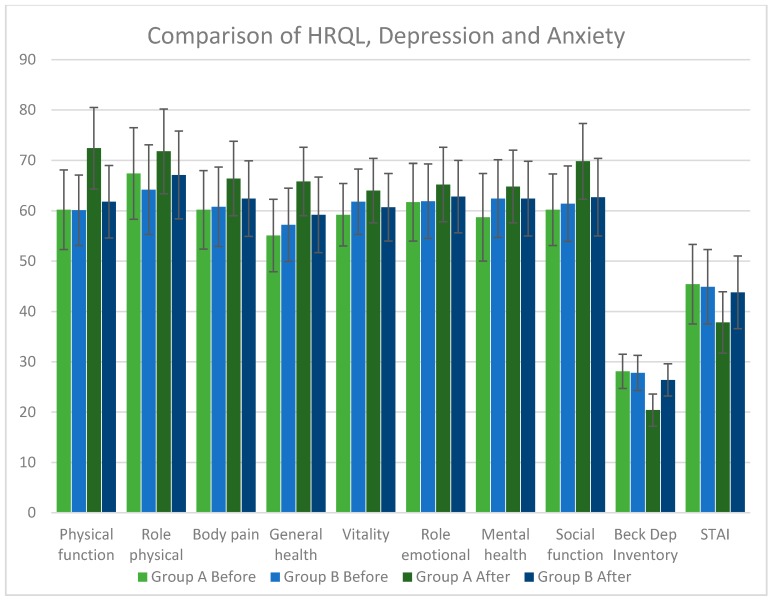
Comparison of health related quality of life (HRQL), Depression, and Anxiety. Group A = Resveratrol Group; Group B = Placebo Group; STAI = State-Trait Anxiety Inventory.

In the comparison between the RV group and the placebo group, we observed a decrease in BDI (*p* < 0.001), in STAI (*p* < 0.001), an improvement in physical function (*p* < 0.001), in role physical (*p* < 0.05), in body pain (*p* < 0.05), in general health (*p* < 0.001), in vitality (*p* < 0.05), and in social function (*p* < 0.001) (Figure 2; Table 3).

### 3.3. Adverse Events

No serious adverse events have been observed in both groups.

## 4. Discussion

In the present study, we tested a cohort of patients with MHE. Our result reveals the presence of moderate depression, anxiety, and a decrease of QoL in subjects with MHE.

The pathogenesis of MHE has been associated with impairment in cerebral energy metabolism with alteration in glucose utilization, glycolysis, and mitochondrial dysfunction. It is thought that impaired mitochondrial function and the down-regulation of the expression of key anti-oxidation enxzymes contribute to an increase in oxidative damage to membrane lipids [27,28,29,30,31,32,33].

It has been demonstrated that RV has remarkable health benefits. One of the major etiopathogenetic factors that are involved in the development of HE is represented by abnormal accumulation in the blood of ammonia. Nonetheless although ammonia seems to have a central role in HE, there is evidence that neurotransmission abnormalities, injury to astrocytes, and microglia activation contribute to the development of HE [34,35]. Hyperammonemia represents a major contributing factor for the development of HE. Recent studies have demonstrated that ammonia exerts effect on many signal transduction pathways, gene expression, and post-translational modifications and influences gene expressions via alteration in micro-RNA expression [36]. Micro-RNAs are small non-coding RNA sequences that play a role in gene silencing at the level of gene transcription as well as translation, and can be regulated by oxidative stress. In RV group we observed a decreased in ammonia serum levels, in depression, in anxiety, and an increase in quality of life (QoL).

RV improves astroglial function and it may play a role in the modulation of glutamatergic metabolism having a cytoprotective action [37,38].

Neurotransmission impairment, caused by an imbalance between the inhibitory and excitatory neurotransmission systems toward a net increase of inhibitory system, is suggested to participate in the pathogenesis of the disease. Several reported findings are consistent with an increase in γ-aminobutiric acid ergic (GABAergic) tone in HE, such as a greater resistance to drug that decreases GABEergic agonists [39,40]. There is also evidence indicating disturbed glutamatergic tone, such as an increase in cortical release of glutamate (Glu) and a decrease in Glu uptake by astrocytes and neurons, leading to high Glu levels in the brain extracellular fluid, lower expression of the astroglia-specific Glu transporter, and a decrease of Glu receptors. An increase in intra-astrocytic glutamine due to the high ammonia levels seems to be a key factor for the development of Alzheimer type II astrocytes.

Microglial activation, which progresses with the development of HE and brain edema, and increases in expression of genes coding for proinflammatory cytokines have been documented in HE.

RV seems to have protective effects in geriatric disorders including Alzheimer’s disease. RV can activate SIRT1, which belong to the sirtuine family of proteins [41]. SIRT1 is involved in epigenetic processes via histones and nonhistone proteins deacetylation and its-regulated pathway is associated with inflammation, cells viability, senescence, and also metabolism.

Murine models demonstrated antipsychotic and anxiolytic properties [42]. Neurological disorders are thought to occur via oxidative and and inflammatory to the CNS [43,44,45].

Studies have reported NF-κB, Akt and STAT3 inhibition by RV. Activation of SIRT-1, a protein recently associated with depressive-like states, inhibits NF-κB via histone deacetylation operated by RV [46,47]. Another SIRT-1 downstream target is represented by AKT, a serine/threonine kinase, which is linked to axonal and neuronal regulation, regeneration and survival [48,49].

RV oxidative and glial inflammation modulation had been demonstrated to prevent ammonia toxicity in astroglial cells.

High levels of ammonia can reduce serotonin and noradrenaline levels in the CNS, resulting in low alertness and attention-associated sleep complaints [10]. Neurobehavioral abnormalities are the major clinical component of HE and have shown to be associated with increased levels of inflammatory cytokines [50]. RV may serve as an antidepressant agent, also exerting anti-inflammatory effects [51,52] and alleviating the consequence of stress with alterations involvement in the immune functions in brain that cause deleterious modifications in the neuronal signaling [53].

The decrease in depression justifies the efficacy of RV in depressed patients with RV.

The pathophysiological mechanisms involved in HE were inflammation, oxidative stress, impaired blood-brain barrier, permeability, neurotoxins, and impaired energy metabolism of the brain [54,55,56,57,58].

Another pathogenic mechanism in HE associated with energy disturbances is the alteration in neurotransmission system, such as the glutamatergic and GABA-ergic systems, resulting in neuronal disinhibition. In vivo preclinical studies had also proposed the involvement of CNS neurotransmitters, including catecholamines, glutamate, GABA, histamine, serotonin, and melatonin [59,60,61,62,63,64,65].

In animal experimental models, RV shows a potent inhibitory monoamine oxidase A and oxidase B and activate serotonine and noradrenaline system [66,67,68,69,70].

There are limited data showing the consequences of RV intake regarding the effects in humans.

This study has several limitations. In fact, we excluded patients with poor HRQoL due to their use of forbidden medications, which could also explain the lack of major changes. We also did not examine confounding factors or potential factors of depressed patients with MHE because the numbers of patients were not statistically powerful.

Such studies need to be increased in number in order to evaluate the role of RV as a modulator of behaviour in MHE patients. However, treatment of minimal HE remains a huge unmet need and a big concerted effort is needed to better defines the multiple pathophysiological mechanisms.

## 5. Conclusions

This clinical trial suggests that Resveratrol, as carnitine derivates and sylibin [71,72,73] is helpful in reducing ammonia serum levels and exhibit sufficient bioavailability at the dosages used without any adverse events. Thus, this study provide a rationale for further testing of resveratrol ub future clinical trials and for studying its effectiveness in treating other neurological disorders.

## Figures and Tables

**Table 1:** Clinical characteristics.

Characteristics	Resveratrol Group 35 pt	Placebo Group 35 pt	*p*
Male/Female	25/10	23/12	N.S
Age (range)	39–60	35–60	N.S
SBP (mmHg)	144.00 ± 18.20	145.00 ± 19.10	N.S
DBP (mmHg)	82.10 ± 10.40	81.80 ± 10.50	N.S
Heart Rate (bpm)	77 ± 8	75 ± 9	N.S
BMI (kg/m^2^)	25.80 ± 2.40	26.10 ± 2.70	N.S
Smokers/No Smokers	15/20	14/21	N.S
Cirrhosis etiology			
Post Hepatitis B	10	11	N.S
Post Hepatitis C	16	14	N.S
Alcoholism	3	2	N.S
Unknown	6	8	N.S
Child-Pugh Class			
Grade A	20	19	N.S
Grade B	15	16	N.S

SBP = Systolic Blood Pressure; DBP = Diastolic Blood Pressure; BMI: body mass index; N.S = not significant.

**Table 2:** Comparison of clinical parameters.

Characteristics	Resveratrol Group 35			Placebo Group 35			After RV→Placebo
	Before	After	*p*	Before	After	*p*	*p*
Urea	51.80 ± 6.10	48.20 ± 6.40	0.019	51.40 ± 5.90	50.20 ± 6.10	N.S	N.S
Ammonia	66.20 ± 7.80	41.40 ± 6.80	<0.001	62.80 ± 7.40	59.70 ± 6.70	N.S	<0.001
AST	87.80 ± 10.70	40.40 ± 9.80	<0.001	80.40 ± 9.80	75.20 ± 8.70	0.022	<0.001
ALT	96.40 ± 11.80	78.40 ± 8.70	<0.001	91.70 ± 10.20	84.20 ± 10.20	0.003	0.013
γ-GT	46.10 ± 6.70	40.40 ± 6.40	<0.001	40.80 ± 6.40	40.50 ± 6.10	N.S	N.S
Prothrombine time	15.40 ± 1.80	15.00 ± 1.20	N.S	15.50 ± 1.70	15.10 ± 1.60	N.S	N.S
Bilirubin	2.00 ± 0.40	2.10 ± 0.50	N.S	2.10 ± 0.40	2.00 ± 0.50	N.S	N.S
Albumin	3.50 ± 0.50	3.70 ± 0.80	N.S	3.60 ± 0.50	3.60 ± 0.70	N.S	N.S

AST = Aspartate aminotranferase; ALT = alanine aminotransferase; γ-GT = gamma-glutamyl transpeptidase; RV = Resveratrol; N.S = not significant.

**Table 3:** Comparison of HRQL, Depression, and Anxiety.

Characteristics	Resveratrol Group		*p*	Placebo Group		*p*	After treatment RV vs. Placebo
	Before	After		Before	After		*p*
Physical function	60.20 ± 7.90	72.40 ± 8.10	<0.001	60.10 ± 7.00	61.80 ± 7.20	N.S	<0.001
Role physical	67.40 ± 9.10	71.80 ± 8.40	N.S	64.20 ± 8.90	67.10 ± 8.70	N.S	0.025
Body pain	60.20 ± 7.80	66.40 ± 7.40	0.001	60.80 ± 7.90	62.40 ± 7.50	N.S	0.028
General health	55.10 ± 7.20	65.80 ± 6.80	<0.001	57.20 ± 7.30	59.20 ± 7.50	N.S	<0.001
Vitality	59.20 ± 6.20	64.00 ± 6.40	0.002	61.80 ± 6.50	60.70 ± 6.70	N.S	0.039
Role emotional	61.70 ± 7.70	65.20 ± 7.40	N.S	61.90 ± 7.40	62.80 ± 7.20	N.S	N.S
Mental health	58.70 ± 8.70	64.80 ± 7.20	0.002	62.40 ± 7.70	62.40 ± 7.40	N.S	N.S
Social function	60.20 ± 7.10	69.80 ± 7.50	<0.001	61.40 ± 7.50	62.70 ± 7.70	N.S	<0.001
Beck Depression Inventory	28.10 ± 3.40	20.40 ± 3.20	<0.001	27.80 ± 3.50	26.40 ± 3.20	N.S	<0.001
STAI	45.40 ± 7.90	37.80 ± 6.10	<0.001	44.90 ± 7.40	43.80 ± 7.20	N.S	<0.001
